# Metallothionein Gene Family in the Sea Urchin *Paracentrotus lividus*: Gene Structure, Differential Expression and Phylogenetic Analysis

**DOI:** 10.3390/ijms18040812

**Published:** 2017-04-12

**Authors:** Maria Antonietta Ragusa, Aldo Nicosia, Salvatore Costa, Angela Cuttitta, Fabrizio Gianguzza

**Affiliations:** 1 Department of Biological, Chemical, and Pharmaceutical Sciences and Technologies, University of Palermo, 90128 Palermo, Italy; salvatore.costa@unipa.it (S.C.); fabrizio.gianguzza@unipa.it (F.G.); 2 Laboratory of Molecular Ecology and Biotechnology, National Research Council-Institute for Marine and Coastal Environment (IAMC-CNR) Detached Unit of Capo Granitola, Torretta Granitola, 91021 Trapani, Italy; aldo.nicosia@iamc.cnr.it (A.N.); angela.cuttitta@iamc.cnr.it (A.C.)

**Keywords:** metallothionein, multigene families, evolution, metal, echinoderms, embryonic development, gene expression

## Abstract

Metallothioneins (MT) are small and cysteine-rich proteins that bind metal ions such as zinc, copper, cadmium, and nickel. In order to shed some light on MT gene structure and evolution, we cloned seven *Paracentrotus lividus* MT genes, comparing them to Echinodermata and Chordata genes. Moreover, we performed a phylogenetic analysis of 32 MTs from different classes of echinoderms and 13 MTs from the most ancient chordates, highlighting the relationships between them. Since MTs have multiple roles in the cells, we performed RT-qPCR and in situ hybridization experiments to understand better MT functions in sea urchin embryos. Results showed that the expression of MTs is regulated throughout development in a cell type-specific manner and in response to various metals. The *MT7* transcript is expressed in all tissues, especially in the stomach and in the intestine of the larva, but it is less metal-responsive. In contrast, *MT8* is ectodermic and rises only at relatively high metal doses. *MT5* and *MT6* expression is highly stimulated by metals in the mesenchyme cells. Our results suggest that the *P. lividus* MT family originated after the speciation events by gene duplications, evolving developmental and environmental sub-functionalization.

## 1. Introduction

Metallothioneins (MTs) represent a superfamily of widespread proteins existing of many organisms, ranging from prokaryotes to vertebrates. The superfamily consists of constitutive and stress-inducible members, with variable masses. It is rich in cysteine (Cys) residues (nearly 30% of their amino acid composition), and the residues constitute the metal–thiolate clusters [1]. MTs possess great affinity for both essential (zinc, copper, selenium) and xenobiotic (cadmium, lead, mercury) metals, binding them through specific Cys-Cys and Cys-Xxx-Cys motifs. Usually, Cys-Cys motifs are located in the C-terminal moiety (also known as α-domain), and Cys-Xxx-Cys motifs map the N-terminal half (or β-domain). MTs are known to exhibit a plethora of biological functions, including protection against metal toxicity, control of oxidative stress and regulation of physiological homeostasis [2,3,4,5]. In addition to their central role as metal scavengers, MTs are also involved in a number of cellular activities, including cell proliferation [6], differentiation [7,8], apoptosis and immune response [9,10]. Finally, MTs have also gained attention in biomedical studies, due to their proposed involvement in cancer and neurological diseases [11,12,13,14,15]. MTs have been classified into different families on the basis of structural features and arrangement of Cys motifs [16,17]. Nevertheless, the large number of MTs discovered so far clearly demonstrates the existence of intermediate isoforms, making the classification of MTs very challenging.

Indeed, mechanisms of duplication, convergence and functional differentiation have created a complex evolutionary history, which is difficult to define [18,19,20].

Through the years, several studies exploring MTs in different organisms (from vertebrates, such as humans, rodents, aves, and amphibians, to invertebrates, such as molluscs, nematodes and insects) have been reported [17].

Deuterostome superphylum diversified around 510 million years ago (Myr), into echinoderms, hemichordates, tunicates and vertebrates. The Echinodermata phylum includes sea stars (Asteroidea), sea urchins and sand dollars (Echinoidea), brittle stars (Ophiuroidea), sea cucumbers (Holothuroidea) and sea lilies (Crinoidea). Thus, Echinoderms represent a very fascinating phylum since they are closely related to Chordates.

Among Echinodermata, sea urchin MT homologues have been identified in *Strongylocentrotus purpuratus* [21], *Lytechinus pictus* [22], *Sterechinus neumayeri* and *Sphaerechinus granularis* [23], revealing an unusual distribution of Cys motifs. Additionally, the three-dimensional structure analysis of *S. purpuratus* MTA revealed that this unusual Cys motif distribution caused an inverted architecture of the α- and β-domains with respect to vertebrate structure [24]. Previously, we reported the identification of five different MT homologues (*PlMT4–8*) from the Mediterranean sea urchin species *Paracentrotus lividus*. Two family members, *PlMT7* and *PlMT8*, are constitutively expressed and upregulated in response to cadmium treatment, whereas *PlMT4*, *PlMT5* and *PlMT6* appear to be specifically switched-on after cadmium exposure [25].

Herein, with the aim of better understanding the evolutionary relationships, functional variety, and the utilization of MTs during development, the gene organisation of *P. lividus MT*s was analysed and their mRNA expression patterns were unveiled. Particularly, we determined the expression profiles and the spatial patterns of *P. lividus MT* transcripts during development and after metal treatments.

Moreover, exploiting the advances in homologues detection and homology protein modelling, theoretical structure calculation methods were applied. Evolutionary perspectives on MTs in deuterostomes were accomplished combining phylogeny and gene features.

## 2. Results

### 2.1. The Metallothionein Genes of P. lividus

The availability of large-scale transcriptional data sets for the Mediterranean sea urchin *P. lividus* allowed us to carry out a transcriptome survey for a comprehensive identification of the MT homologues. We performed BLASTN and TBLASTN searches using MT cDNA sequences previously cloned as queries [25] and a clustering analysis of MT expressed sequence tags (EST) retrieved. No MT4, MT5 or MT6 sequences were found in the databases, confirming their low expression. Collectively, two *MT7* transcript populations differing in length and three *MT8* different populations were retrieved. Their identification was checked manually and the matching sequences were reconfirmed by comparative analysis. These results suggest that *MT7* transcripts may derive from a single gene by alternative splicing or multiple polyadenylation signals or even from two different genes. Moreover, it is possible to hypothesise the presence of at least three *MT8* genes.

In order to identify and isolate the expressed *MT* genes, total genomic DNA from *P. lividus* sperm was extracted and amplified using primer pairs selected as described in Materials and Methods. The amplified products were cloned, sequenced and analysed. Four genomic clones, coding for MT4, MT5, MT6, MT7 and three diverse clones corresponding to MT8 (named MT8a, b, c) were obtained.

**Figure 1:**
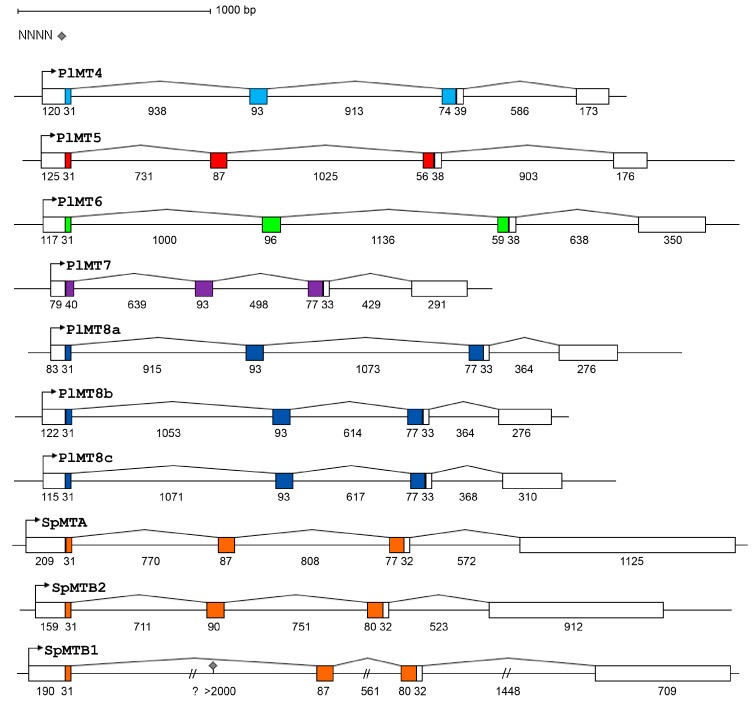
Schematic gene structures of the *Paracentrotus lividus* and *Strongylocentrotus purpuratus* metallothioneins (*MTs*; drawn to scale). The bent arrows indicate the putative transcription start sites (TSS). Numbers under schemes indicate base pair numbers of exons and introns; boxes represent exons: white boxes indicate untranslated regions, and coding regions are coloured. The grey diamond indicates one N stretch in the *SpMTB1* intron.

The comparison between cDNA and genomic sequences revealed that the transcription units are composed by four exons interrupted by three introns and are different in length. The first two introns interrupt the coding sequence after the first nucleotide of the codon (phase-1), the last intron is located in the 3′ UTR. The gene structures of the *P. lividus MT* genes are represented in Figure 1. All of them possess canonical splicing sites, identified at 5′-end by GT and at 3′-end by AG consensus sequences. Moreover, a comparative analysis between *P. lividus* and *S. purpuratus MT* genes (Strongylocentrotid diverged 35–50 Myr from the Parechinidae [26]) showed that all possess the same structure [27]. Nevertheless, intron lengths and sequences are different between all homologous genes.

In silico predictions showed two polyadenylation sites in the *MT7* gene (score 0.876 and 0.898) which could explain the presence of two *MT7* mRNA species different in length during embryo development.

*MT8b* and *MT8c* showed approximately the same length and 94% identity. Both genes contain 136 additional bps in the first intron and a 476-bp deletion in the second intron with respect to *MT8a*. In alignable sequences, the identity between *MT8a* and *MT8b* is 97%, higher than the identity with *MT8c* (93%). The *MT8a* and *MT8b*-expressed sequences show 98% of identity between them and 95% with *MT8c*, differing for the presence of a simple AT-rich region in the 3′ UTR.

### 2.2. Predicted 3D Structural Model of P. lividus MTs

On the basis of the computational analysis, we determined the key features of MT homologues in *P. lividus*. As previously highlighted [25], only MT7 and MT8 follow the MT family 4 rule (Echinoidea: IPR001396; [17]): P-D-x-K-C-[V,F]-C-C-x(5)-C-x-C-x(4)-C-C-x(4)-C-C-x(4,6)-C-C located near the N terminus. Other isoforms instead have divergent amino acid sequences and in particular MT4 and MT6 have a cysteine pattern that is slightly different.

**Figure 2:**
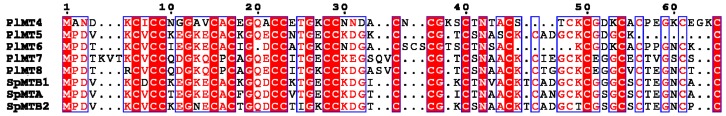
Multiple sequence alignment (MSA) of *P. lividus* and *S. purpuratus* MT sequences. Identities and conservative substitutions are in red font. Red shading represents identity among all sequences. Dots denote gaps. Non-conservative substitutions are in black font.

The MTs are organized in a N-domain and a C-domain bearing a pattern of conserved Cys residues required for binding bivalent metal ions [17]. As occurred in *S. purpuratus MTs*, Cys-Cys motifs, typical of the C-domain in mammals, are located in the N-domain. Multiple sequence alignment (MSA) analysis of *P. lividus* and *S. purpuratus* MTs (Figure 2) showed that, in addition to conserved Cys pattern, the accepted amino acids substitutions do not always possess similar physical chemical features. We argue that such changes might not support the same structure and could alter biochemical properties, allowing MT involvement in different pathways.

In order to obtain some indication on structure changes, we computed the secondary elements and derived the 3D structures of *P. lividus* MTs (PlMTs). Different templates were selected to model the Mediterranean sea urchin MTs, on the basis of heuristics to maximise confidence, percentage identity and alignment coverage. If required, insertions were modelled ab initio. The generated models were validated by assessing Ramachandran plot analysis and the percentage of residues in the favoured/allowed region ranged from 91% to 96%. In a manner similar to those described in previous studies, PlMTs mainly consisted of coils and turns; while different numbers of helical structures were computed (three α-helices in MT4, MT6 and MT8, two and four α-helices in MT5 and MT7, respectively).

**Figure 3:**
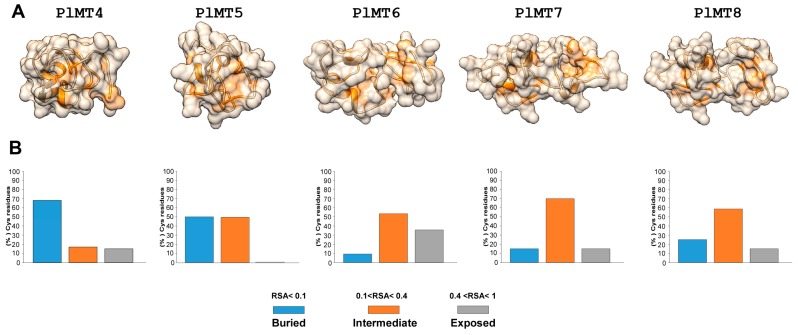
Ribbon diagrams and surface representations of PlMTs. (**A**) General overview of the 5 PlMTs generated by homology modelling with cysteine residues labelled in orange. As far as possible, N-terminal is on the left and C-terminal is on the right; (**B**) Relative solvent accessibility (RSA) calculated as percentage of corresponding cysteine residues in each protein.

Generally, the global structure of each protein resembled that of MTs from other organisms: two domains connected by a flexible hinge. However, MT4 and MT5 proteins appeared to be more compact than MT6, 7 and 8 (Figure 3). The relative solvent accessibility (RSA) of the Cys residues in the folded protein was also calculated. Based on RSA values, sea urchin MTs exhibited a similar pattern of solvent accessibility in the Cys, as such residues were found to mainly adopt a buried (RSA < 0.1) or intermediate (0.1 < RSA < 0.4) conformation. Conversely, exposed and highly exposed residues (0.4 < RSA < 1 and RSA > 1 respectively) were found to be strongly underrepresented (Figure 3).

According to the structural features of *S. purpuratus* MTs (SpMTs) [24] and in silico protein–ligand binding site recognition, possible metal–thiolate cluster structures of PlMTs were predicted. The absence of specific Cys residues in the C-domain of MT4, 5 and 6 as well as the presence of an additional Cys residue in the N-domain of MT6 raise the possibility that these homologues were likely prone to accept variations in metal binding without modifications of the total number of bonded divalent cations. Elsewhere, the N- and C-domains of these homologues may likely encompass different metal–thiolate clusters.

**Figure 4:**
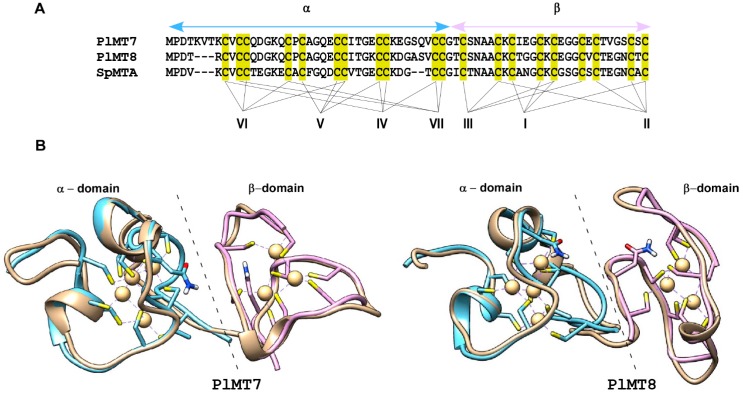
Metal–thiolate cluster analysis and structural similarities between PlMT7, PlMT8 and SpMTA. (**A**) Cluster connectivities between the cysteine thiolate groups and the metal ions in the α- and β-domains of the sea urchin MTs. Cys residues are boxed in yellow, while the metal ions are specified by roman numerals; (**B**) Superposition of the 3D structures of PlMT7 and PlMT8 with SpMTA. Proteins are in ribbon representation. The Cys residues are in stick representation; a Gln residue is also shown. The *P. lividus* proteins are in ivory, the α-domain of SpMTA is shown in cyan and the β-domain is in violet. Superposition was created and rendered using Chimera package.

As shown in Figure 4, four and three metal–thiolate clusters were identified in the N- and C-domain respectively of MT7 and MT8. The cluster connectivities between the cysteine thiolate groups and the metal ions in the N- and C-domains of MT7 and MT8 were compared with the well-defined SpMTA. Thus, algorithms to superimpose the polypeptide backbones from these homologues were applied and analogous spatial arrangements of Cys residues among structures and related metal–sulfur clusters were retrieved (Figure 4).

It should be noted that computational prediction of the metal binding sites in *P. lividus* as well as in *S. purpuratus* MTs suggests alternative thiolate clusters, composed by Cys encompassing residues from both the N- and C-domain. Thus, it could be hypothesised that a wide range of conformational states and metal interactions between domains could be supported.

### 2.3. Phylogenetic Analysis of Deuterostome MTs

**Figure 5:**
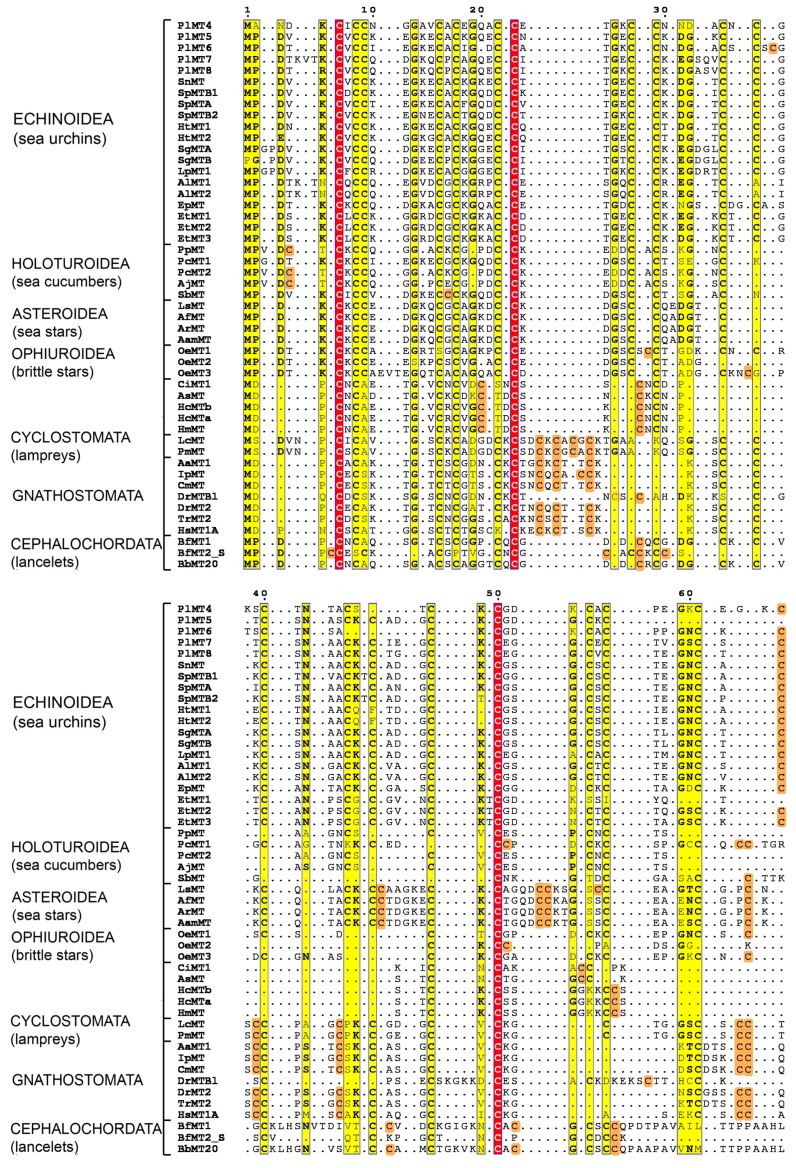
MSA of MT protein sequences. Conservation score higher than 0.5 is highlighted in yellow. Unaligned cysteines are in orange. Cysteines aligned in all sequences are highlighted in red. Dots denote gaps. Species acronyms, taxonomy and sequence IDs are indicated in Table 1.

**Figure 6:**
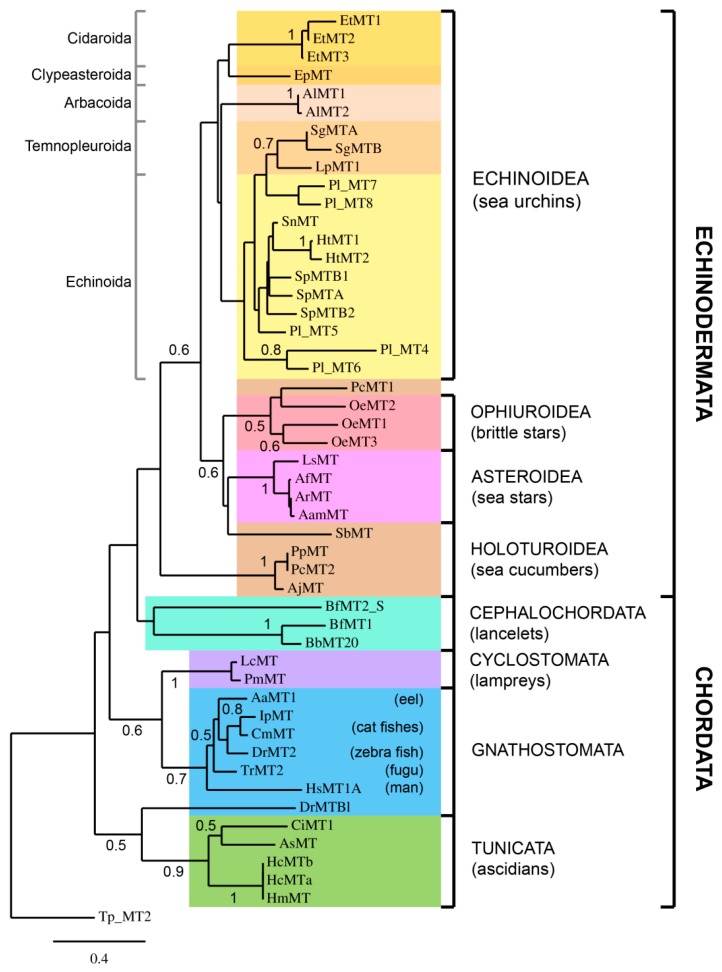
Amino acid sequence-based neighbour-joining tree. Species acronyms and sequence IDs are indicated in Table 1. The values at nodes indicate bootstrap support greater than 50%. *Tetrahymena pyriformis* was used as outgroup to root the tree. Branch lengths are drawn to a scale of amino acid substitutions per site.

In order to study metallothionein evolution in deuterostomes, annotated protein sequences were retrieved from databases. Moreover, when not already annotated, MT sequences were searched by similarity in transcriptome databases (Table 1). All available transcriptomes of Echinodermata species were considered, including Asteroidea (sea stars), Ophiuroidea (brittle stars), and Holothuroidea (sea cucumbers). Moreover, for understanding and gaining insight into the evolutionary trends of the MTs, phylogenetically interesting species belonging to non-vertebrate chordates as amphioxus and ascidians were considered [28,29,30]. Additionally, jawless vertebrates (agnates/Cyclostomata), representatives of an ancient vertebrate lineage, and relatively early diverging teleost fishes, like eels and cat fishes were selected. Also two well-studied fishes, the Japanese tiger puffer and the zebra-fish, and a human MT were chosen [31,32]. Unfortunately, no acorn worm (Hemichordata) nor sea lily (Crinoidea) MT sequences were found. The MSA between MTs from these different classes is shown in Figure 5. The alignment was also used to construct the phylogenetic tree shown in Figure 6. As an outgroup, an MT from the ciliate protozoa *Tetrahymena pyriformis* was selected. Tetrahymena MTs constitute an excellent example of an MT subfamily well studied in terms of molecular genetics and protein levels [33,34,35]. This subfamily is considerably divergent from the deuterostome paradigm, so it is unambiguously outside the clade of interest in this phylogenetic study. Nevertheless, they can be successfully aligned to sequences from the ingroup. Since the three MT isoforms identified in *T. pyriformis* are longer than deuterostome ones [36,37,38], we selected the shortest one (MT-2) as the outgroup.

The phylogenetic analysis of echinoderm MT sequences suggested that many events of gene duplication have occurred independently in different species. For each species the sequence divergence is very different, suggesting that gene duplication occurred after speciations and at different times. MTs of echinoderms exhibit high sequence heterogeneity, both among them and in relation to the vertebrate peptides, and PlMTs appear the most divergent. MTs of *Eucidaris tribuloides*, a representative cidaroid, branch apart from other sea urchin sequences, confirming cidaroids are the basal group within the class [39]. *Echinarachnius parma*, a representative clypeasteroida, results in the cidaroid sister branch.

Even though the inter-relationships of the classic deuterostome phyla appear to be resolved, the intra-relationships within each of the phyla have been troublesome and not completely concluded [40]. The analysis of the different classes of Echinodermata MTs suggests that ophiuroid MTs were more closely related to asteroid than to the echinozoan MTs and, in turn, to the holoturian MTs.

As already reported, the MT sequences of tunicates and amphioxus show a low identity with other deuterostome MTs, not being directly alignable to the isoforms of other classes [28,30]. Thus, Branchiostoma MTs appear in a separate tree branch with respect to the two tunicates and group with the echinoderm isoforms at a greater distance of the vertebrate counterparts. These MTs are the most similar to MTs of molluscs (data not shown). Sea squirt MTs are the shortest MTs so far identified in deuterostomes and are clearly separated from other Chordata and Echinodermata MTs. Finally, the MTs of lampreys, representatives of an ancient vertebrate lineage that diverged from our own ~500 Myr, as expected, appear the nearest related to other vertebrata MTs.

In order to analyse also *MT* gene structure evolution, new BLAST searches in genomic databases were performed using *MT* mRNA sequences as queries (Table 1). Genomic sequences of already annotated *MT* genes were also retrieved from databases. Novel annotated genes from selected genome draft sequences were compared to already known *MT* genes.

**Figure 7:**
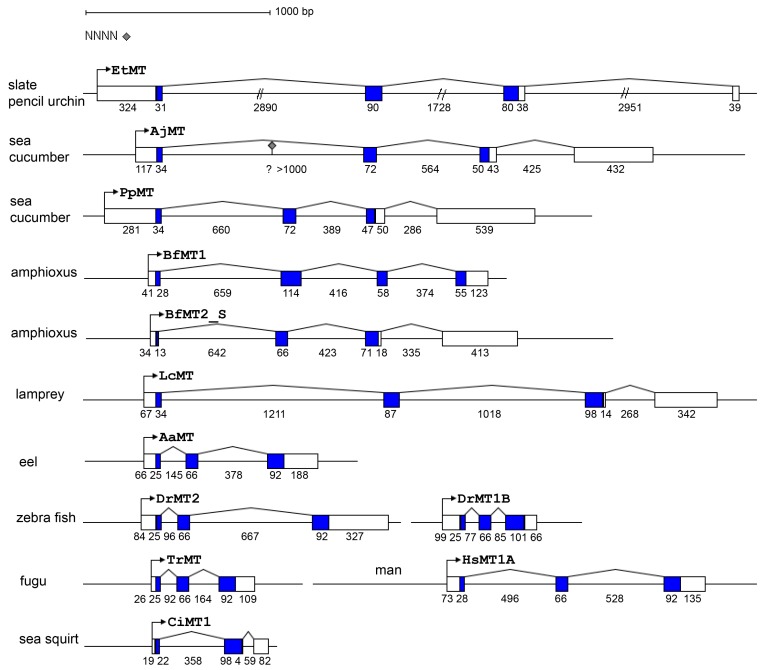
*MT* gene structures of echinoderms and chordates (drawn to scale). The bent arrows indicate the putative TSSs. Numbers under schemes indicate base pair numbers of exons and introns; boxes represent exons: white boxes indicate untranslated regions, and coding regions are coloured. The grey diamond indicates one N stretch in the *AjMT* intron.

For the first time, *MT* genes of the slate pencil urchin of the Cidaroida order (*E. tribuloides*), two sea cucumbers (*Apostichopus japonicus* and *Parasthicopus parvimensis*), a lamprey (*Lethenteron camtschaticum*) and an eel (*Anguilla anguilla*) were characterized. Gene structures are depicted in Figure 7 and show clearly the high variability of intron lengths, the longest being the Cidaroida *E. tribuloides* gene and the shortest the fish genes. All the introns that interrupt the coding regions of the selected genes are in phase-1 and all the coding regions with the exclusion of the ascidian one [28] are interrupted by two introns. Interestingly, the lamprey gene contains also the intron in the 3′ UTR like echinoderm and ascidian ones. As already reported, lancelet (*Branchiostoma floridae*) gene structures are in some way unique, indeed *BfMT1* lacks the intron in the 3′ UTR and the *BfMT2* first intron is located at only 4 codons from the ATG [30]. Thus, also gene structures reflect divergence in coding sequences.

### 2.4. Expression of P. lividus MT Family Genes during Development and after Metal Treatments

#### 2.4.1. Physiological Expression during Embryogenesis

**Figure 8:**
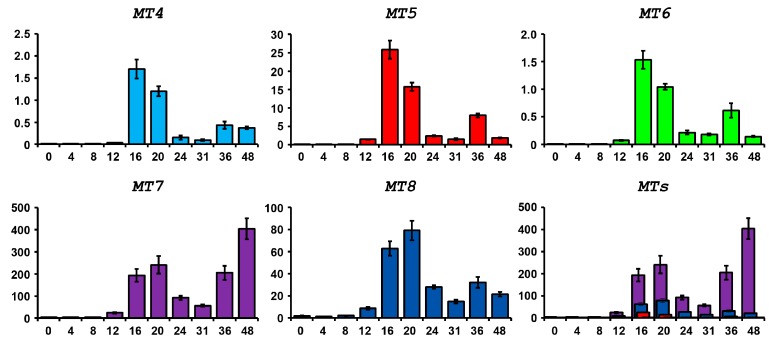
*MT* expression during normal development. RT-qPCR results are shown in arbitrary units (AU) with respect to 18S RNA. The last panel (*MT*s) show the superposition of the other profiles, in order to display *MT* relative expression.

As already reported [25], embryos growing in normal conditions at the gastrula stage express *MT7* and *MT8* transcripts at high levels. Conversely, *MT4*, *MT5* and *MT6* are expressed at low levels. In order to study metallothionein expression throughout *P. lividus* embryo development, reverse transcription and quantitative (real time) PCR (RT-qPCR) experiments were performed. During development under normal conditions, *MT4*, *MT5* and *MT6* are expressed at very low levels, however levels rise significantly between 16 and 20 h and then decrease at the later stages (31 h), showing a second intensification peak at 36 h (mainly *MT5* and *MT6*). The *MT8* expression profile during development is similar, even if its average level is much higher. Conversely, *MT7* (the most expressed MT) increases not only during the beginning of gastrulation but also at the later stages, reaching a peak at the pluteus stage (Figure 8).

#### 2.4.2. Expression after Metal Exposure

Metals, such as copper, zinc, lead, mercury and nickel, can all act as poisons or teratogens, causing deformities or deaths in many organisms exposed to them. It is well known that sea urchins are particularly susceptible to environmental conditions and respond readily [41,42]. It is widely accepted that MTs are involved in many functional roles, ranging from toxic metal protection and physiological homeostasis, to free-radical scavenging or oxidative stress protection and antiapoptotic defences.

In order to extend the knowledge on the functional role of *P. lividus* MTs, we studied their mRNA expression in response to metal exposure. Embryos were treated during development with different metals: zinc (Zn) and copper (Cu), two essential metals, and a metal with intermediate characteristics, nickel (Ni). Moreover, since we already studied *PlMT* expression in response to cadmium exposure [25], we chose lead (Pb) as a nonessential metal with recognized toxicity. Embryos were continuously treated with metal concentrations ranging from 10^−8^ to 10^−4^ M, except the Cu treatment, that ranged from 10^−8^ to 10^−5^ M, because embryos exposed to 10^−4^ M CuSO_4_ did not survive. It is in fact known that copper is the most toxic metal to developing embryos and larvae of sea urchin [43,44].

**Figure 9:**
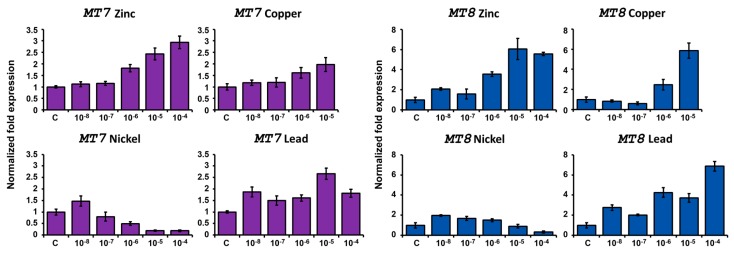
Expression of constitutive *MTs* after metal exposure. RT-qPCR results performed on RNA extracted from 24 h embryos treated with zinc, copper, nickel or lead. C: controls grown in millipore-filtered seawater (MFSW). Metal treatments were performed with concentrations ranging from 10^−8^ to 10^−4^ M. Copper treatments ranged from 10^−8^ to 10^−5^ M.

**Figure 10:**
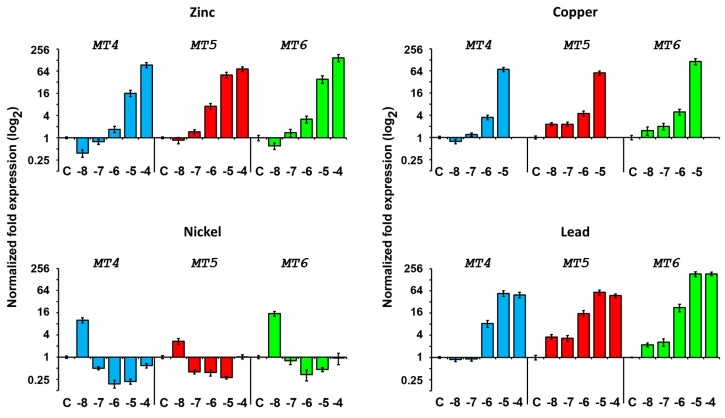
Expression of induced *MTs* after metal exposure. RT-qPCR results performed on RNA extracted from 24 h embryos treated with zinc, copper, nickel or lead. C: controls (MFSW). Metal treatments were performed with concentrations ranging from 10^−8^ (−8) to 10^−4^ M (−4). Copper treatments ranged from 10^−8^ (−8) to 10^−5^ M (−5). These graphics are in log_2_ scale.

The development of treated and untreated embryos was observed under microscope until 48 h post fertilization. In order to study the response caused by metals, RNAs were extracted from embryos at 24 h of development, corresponding to gastrula stage in normal conditions, when all *MT* transcripts are expressed at relatively low levels. RNA were used in RT-qPCR experiments for testing *MT* transcript relative quantities (Figure 9 and Figure 10).

##### Zinc Treatment

According to previous studies, low levels of zinc had no effect on the development of the sea urchin embryos, however higher concentrations caused abnormal development, animalizing the embryos [45,46].

RT-qPCR results showed that embryo exposure up to 10^−7^ M Zn does not grossly affect *MT7* levels. Conversely, higher concentrations cause a progressive increase in level of this transcript. It is worth noting that *MT7* levels are high also in embryos developed in normal conditions and that the 3-fold upregulation caused by zinc is the highest observed. Instead, *MT8* expression is affected also at the lower Zn concentration tested, rising until 10^−5^ M and then reaching a plateau (6-fold with respect to controls). The low Zn concentration (10^−8^ M) causes a decrease of induced *MTs* (especially *MT4*), conversely, higher concentrations stimulate gene expression progressively (>100×).

##### Copper Treatment

Cu has been shown to cause multiple deformities. Most of these abnormalities occurred at the gastrulation stage of development [47]. At low concentrations, radialized plutei larvae were observed. Developmental effects on the skeleton, consisting of an underdeveloped skeleton with no spicule formation or multiple triradiate spicules and elongated spicules were observed [48]. Despite these abnormalities, *MT* levels are affected only at relatively high concentrations. Indeed, all *MTs* except *MT5* increase in their levels between 10^−7^ and 10^−6^ M. *MT5* responds first, rising readily at the lowest tested concentration (10^−8^ M).

##### Nickel Treatment

Ni ventralizes the ectoderm; as a consequence, the arrangement of primary mesenchyme cells (PMCs) in the blastocoel is altered and the number of spicule rudiments is increased [49,50]. All *MTs* increase at the lower metal concentration and decrease at all other tested concentrations. It is worth noting that Ni influences very little *MT* transcript levels with respect to other metals.

##### Lead Treatment

Pb is considered less toxic for *P. lividus* embryos than Cu [43,51], on the other hand its bioaccumulation proceeds faster than Cu [52]. When embryos were raised continuously in Pb, both the patterning of the PMC ring and the expression of *MTs* were altered, having a pattern very similar to zinc response. However, maximal levels of transcripts were reached with the 10^−5^ M Pb treatment for all *MTs* except for *MT8*. *MT8* indeed peaked at 10^−4^ M Pb.

### 2.5. Spatial Patterns of MT Gene Expression

In order to complete the *MT* expression overview and to shed some light on MT function/utilization during normal development and under metal exposure, we performed whole-mount in situ hybridization experiments (WMISH). Because the effects of metal treatments appear clearly visible during gastrulation, developmental stages corresponding to gastrula and pluteus were used in WMISH to define the *MT* expression in the different embryo tissues and cell types.

**Figure 11:**
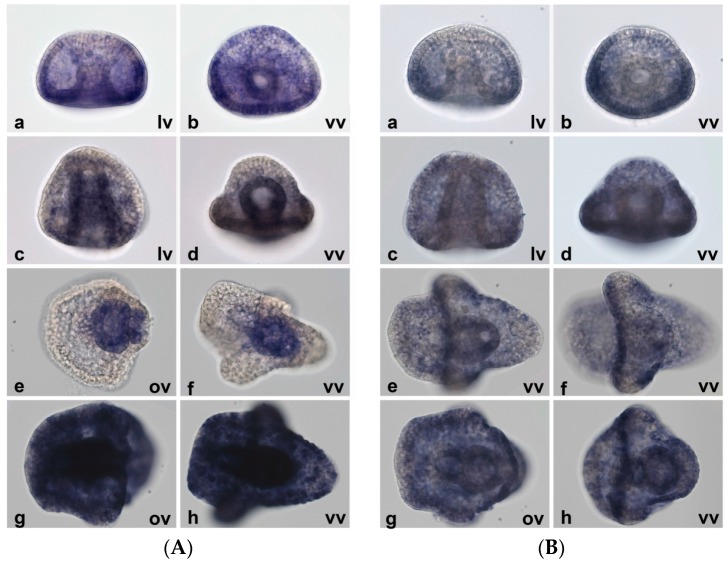
Localization of constitutive *MTs* during normal development. Whole-mount in situ hybridization experiments (WMISH) results, 1 h of staining. (**A**) *MT7*; (**B**) *MT8*: (**a**,**b**) gastrula stage (24 h); (**c**,**d**) prisma stage (31 h); (**e**–**h**) pluteus stage (48 h); (**e**,**f**) in the (**A**) panel, visualization after 15 min of staining; (**e**,**f**) in the (**B**) panel, different focuses of the same embryo are shown. 40× magnification. lv: lateral view; vv: ventral view; ov: oral view.

As shown in Figure 11A, the embryos grown in uncontaminated seawater display robust *MT7* expression, indeed staining times of only 15 min were sufficient to detect the transcript. At the gastrula stage, *MT7* is localized principally in the endomesoderm, in the vegetal pole. Progressively, it becomes heavily expressed in the endoderm during archenteron specialization in midgut and hindgut and then in stomach and intestine at the pluteus stage. Besides this localization at a very high level, *MT7* is also expressed in all other tissues, as shown after 1 h of staining (Figure 11A(g,h)).

In contrast, at the gastrula stage *MT8* appears localized in the oral ectoderm and in the ventral region of the ciliary band. It is not expressed in the archenteron, not even at the later stages of development. At the pluteus stage, it is mainly localized in a narrow strip of cells between the anal arms of the larva (the boundary between oral and aboral ectoderm) and lightly in the oral ectoderm (Figure 11B).

**Figure 12:**
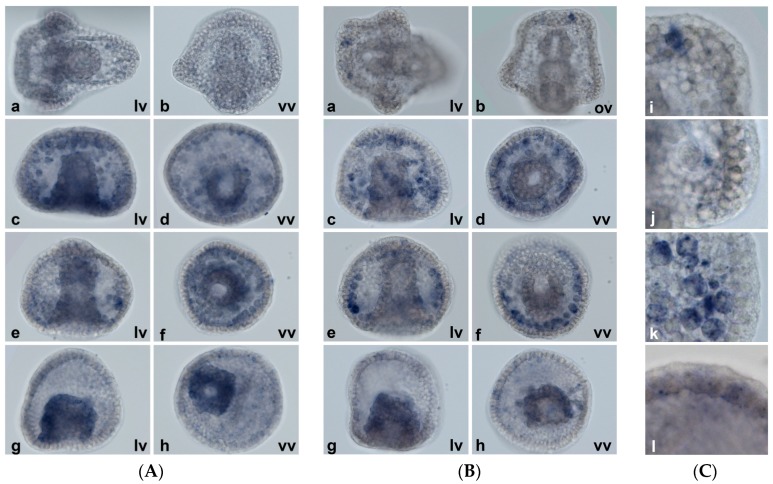
Localization of induced *MTs* at 48 h of normal development and after metal exposure. WMISH results. (**A**) *MT5*; (**B**) *MT6*; (**C**) *MT6* details: (**a**,**b**) untreated embryos, pluteus stage (48 h) after 3 h of staining; (**c**,**d**) 10^−7^ M CdCl_2_ prisma stage (31 h); (**e**) and (**f**) 10^−6^ M CuSO_4_ prisma stage (31 h); (**g**,**h**) 10^−7^ M ZnCl_2_ prisma stage (31 h); (**C**) enlargements of the (**B**) panel: (**i**,**j**) enlargement of (**b**), untreated embryos, pluteus stage (48 h); (**k**) 10^−7^ M CdCl_2_ prisma stage (31 h) enlargement of (**e**) (different focus plan); (**l**) 10^−7^ M ZnCl_2_ prisma stage (31 h) enlargement of (**g**) (different focus plan). 40× magnification. lv: lateral view; vv: ventral view; ov: oral view.

The levels of inducible *MTs* are very low in control embryos, thus they are usually undetectable by WMISH. At the pluteus stage (that corresponds to a peak of expression) *MT5* does not appear (see Figure 12A(a,b)); conversely, *MT6* is detectable in couples of cells at the tips of the elongating anterolateral and postoral skeletal rods, while no expression is visible in cells of the apex (Figure 12B,C). *MT4* is not detected at any stage, presumably because its messages, that are expressed at very low levels, are widely dispersed (data not shown).

Taking into account the localization of *MTs* transcripts in control embryos, we decided to perform WMISH experiments on embryos exposed to metals (10^−7^ M CdCl_2_, 10^−7^ M ZnCl_2_ or 10^−6^ M CuSO_4_) that disrupt development, affecting oral/aboral axis formation. Consequently, PMCs are not directed to their correct arrangement in a posterior ring around the blastopore with ventrolateral clusters, thus impairing skeletogenesis [49,50]. In cadmium and copper treated embryos, after their ingression into the blastocoel, PMCs migrate into a ring pattern within the blastocoel. In Zn-treated embryos, ectoderm territory is expanded and mesenchyme cells do not detach from the endoderm and take up position covering the entire abnormal archenteron.

**Figure 13:**
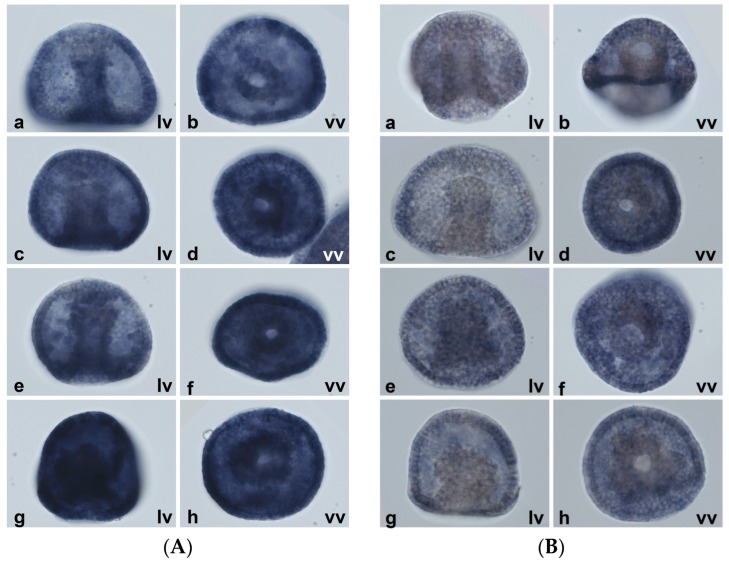
Localization of constitutive *MTs* after metal exposure. WMISH results at 31 h (prisma stage in the controls). (**A**) *MT7*; (**B**) *MT8*; (**a**,**b**) untreated embryos (controls); (**c**,**d**) 10^−7^ M CdCl_2_; (**e**,**f**) 10^−6^ M CuSO_4_; (**g**,**h**) 10^−7^ M ZnCl_2_. 15 min of staining. 40× magnification. lv: lateral view; vv: ventral view.

WMISH showed that in embryos treated with each metal, *MT7* is overexpressed throughout the embryo (Figure 13A). *MT8* loses its localization in the oral ectoderm and in the ventral ciliary band, and it becomes uniformly distributed in the unspecialized ectoderm (Figure 13B). In any case, it is never detectable in the endoderm.

Differently from constitutive isoforms, in the embryos treated with metals *MT5* and *MT6* are expressed in the mesenchyme cells, no matter where they are arranged in the blastocoel (Figure 12). *MT5* is also slightly expressed in the hindgut. Interestingly, at higher magnification it appears that the hybridization signals are not homogeneously distributed within the cells (Figure 12C(k)). Additionally, in the blastocoel surface of some ectodermal cells a few punctiform signals appear (Figure 12C(l)).

## 3. Discussion

In *P. lividus* embryos, the first MTs were described by Scudiero et al. [53] and the first cDNA was obtained in the Matranga lab, here referred to as *Pl-MT1* [54]. In a previous work we isolated five cDNAs, one of them (*MT8*) with a sequence very similar to *Pl-MT1* [25].

Here, we studied the *MT* multigene family of the Mediterranean sea urchin species of Parechinidae and compared it to that of other echinoderms and early chordates. The *P. lividus* genome harbours at least seven expressed metallothionein genes that we characterized. By a transcriptome-wide survey, for the first time, we identified expressed RNA sequences that encode previously uncharacterized MTs. Moreover, we described the *MT* gene structures of a slate pencil urchin, two sea cucumbers, a lamprey and an eel. All the echinoderm *MT* genes share the same intron–exon organization. Interestingly, the lamprey gene also displays the same structure, indicating that the last common ancestor of vertebrates and echinoderms could have the intron in the 3′ UTR and that this intron would have been lost after the branching of the jawless fishes (Cyclostomata) and the jawed vertebrates (Gnathostomata).

As the slate pencil urchin *E. tribuloides*, a member of the basal echinoid order Cidaroida, shows the longest *MT* gene here described (and the fishes the shortest ones), we can infer that *MT* gene structure was characterised by a decrease in intron length during evolution. It is known that large introns can contribute to proteome diversity by facilitating alternative splicing [55]; on the other hand, during evolution eukaryotes seem to have undergone extensive intron loss, favouring high expression levels. It has also been described that genes that are rapidly regulated during stress contain significantly reduced intron numbers and length [56]. Since *MT* genes are highly expressed and/or rapidly activated after stimuli, these theses could fit to the observations we made on the *MT* intron evolution.

Transcriptome searches revealed up to three paralogs in echinoderm species examined and one or two in chordates. It is worth noting that this analysis (in transcriptomes of organisms grown in normal conditions) does not allow finding other possible *MTs* not constitutively expressed and induced by a stimulus. It could be interesting to know the complete MT families of other Echinodermata, in order to infer their possible orthology. Incidentally, the fact that the genes coding for metallothioneins are not ordinarily annotated by gene-finding programs is not surprising, because the coding regions are not only very small but also interrupted by large introns. In any case, the identified “constitutive” SpMTs are very similar among them, as *P. lividus* constitutive isoforms; conversely, *P. lividus*-induced MTs diverge particularly in the C-terminal domain. Since SpMTA 3D structure is known, by homology modelling we modelled the PlMT structures, revealing that, as expected, MT7 and MT8 show a 3D structure similar to SpMTA. Differently, MT4 and MT5 appear to be more compact than MT6, 7 and 8 with less distinguishable α- and β-domains.

The phylogenetic analysis of protein sequences suggests that ophiuroids were more closely related to asteroids than to the echinozoans (the asterozoan hypothesis reviewed in [57]). Moreover, our results confirm the recent estimate of chordate intra-relationships: that chordates are monophyletic and that vertebrates share a last common ancestor with urochordates to the exclusion of cephalochordates [30,40]. Indeed, as shown before [30], phylogenetic studies places amphioxus MTs in the same echinoderm branch.

PlMTs show not only a high degree of evolutionary sequence and structure differentiation but also a high degree of functional differentiation: *PlMT* genes are differently expressed, both quantitatively and spatially, and respond distinctively to metal-dependent transcriptional activation. Indeed, *MT7* and *MT8* are expressed under physiological conditions at different levels and in different tissues (see also [58]), besides they respond relatively little and differently from each other to metal overload.

Conversely, *MT4*, *5* and *6* are expressed at basal levels during normal development and their expression bursts under metal exposure. Differences can also be found between these induced isoforms; indeed, *MT4* is probably widespread in the embryo, while *MT5* and *MT6* are expressed in the mesodermal cells. In particular, *MT6* is highly expressed under normal conditions in a very low cell numbers, at least at the pluteus stage. Its localization resembles that of *Lvmsp130* [59] in cells at the tip of the skeletal rods, even though we could not observe expressing cells in the scheitel. Probably, as with *Lvmsp130*, the reduction in *MT6* mRNA levels at later stages could be due to a minor number of expressing cells (only a subset of the PMCs).

Interestingly, even taking into account their difference in quantity, the *MTs* grossly exhibit the same level variations during development. This could be related to the fluctuation in the overall transcription during embryogenesis; however, the induced *MTs* peak at 16 h after fertilization, before the other *MTs* and just when PMC ingression occurs. Thus, it is reasonable to suppose a role for these MTs in PMC differentiation and/or activity.

It is known that both Pb and Zn render their toxic effects through disruption of Ca homeostasis [60,61], impairing calcium accumulation which is necessary for spicule deposition. Indeed, the sea urchin skeleton is composed of the calcium carbonate mineral calcite and numerous associated proteins [62]. Thus, treatment with metals impairs skeletogenesis, although in different manners, directly affecting the biomineralization process and/or disrupting the oral aboral axis and then PMC localization [49,50].

Unexpectedly, *MT5* and *MT6* WMISH signals do not seem uniform in the PMCs. This could be a technical artefact; alternatively, these transcripts could be accumulated in vesicles. Indeed, Beniash et al. [63] showed that the PMCs contain electron-dense granules (named calcein puncta) which correspond to calcium-rich vesicles that contain nanospheres of amorphous calcium carbonate which then partially transforms into calcite during spicule mineralization. The calcein puncta are distributed widely all over the embryo, not only in the primary mesenchyme cells but also in the surface of the epithelial cells [64].

All this, and also the puncta observed in the surface of the epithelial cells, suggests that *MT5* and *MT6* could be accumulated in the calcein puncta. Thus, *MT* induction and mRNA storage just in PMC puncta after metal exposure corroborate the hypothesis of a protective role during calcium deposition in skeletogenesis.

Although it is not possible to infer orthology from MT sequences of sea urchins, it should be remembered that *S. purpuratus MTs* also exhibit different spatial, temporal and quantitative patterns of expression [21,65,66]. Hence, *SpMTA* is transcribed in ectodermal tissues, such as *PlMT8*, while *SpMTB* is expressed in both the ectodermal and endodermal structures, such as *PlMT7*. However, quantitative patterns are inverted. Indeed, under physiological conditions *SpMTA* mRNA levels reach up to 10 times those of *SpMTB*, and *PlMT8* levels are five times lower than *PlMT7*. Moreover, under metal overload, *SpMTB* and *PlMT7* expression is further induced, so that *SpMTB* and *PlMT7* achieve levels similar to those of *PlMT8* and *SpMTA*, respectively.

In summary, *P. lividus* MTs show a high polymorphism both in sequence/structure and expression pattern. In the light of our findings it seems reasonable to consider MT7 and MT8 as the major variants associated with physiological functions, playing their major roles in metal homeostasis and redox activity in ecto-, meso- and endo-dermal tissues. On the other hand, a heavy metal detoxification role can be attributed to MT4, 5, and 6, particularly important in mesenchyme cells for the skeletogenic pathway. However, since differences not only between the two classes occur, each isoform would correspond to a more definite physiological function. In fact, it was also recently demonstrated that induced *PlMT*s are regulated in dissimilar manners by oxylipins, the polyunsaturated aldehydes produced by diatoms [67,68]. Also, manganese exposure caused a slight up-regulation of *MT5* and *MT8*, and a down-regulation of *MT6* expression at the pluteus stage [69,70].

Thus, it will be interesting to obtain information on the transcriptional mechanisms that control basal and induced *MT* gene expression in sea urchin embryogenesis, in physiological and stress conditions.

## 4. Materials and Methods

### 4.1. Database Analysis, Cloning and Sequence Analysis of MT Genes

*P. lividus MT* cDNA sequences previously identified [25] were used as queries in a genome-wide basic local alignment search tool (BLAST) [71] screening of genomic scaffolds in the *P. lividus* (genomic draft) database (v2.0, available at http://octopus.obs-vlfr.fr/index.php). These partial genomic sequences were essential to design oligonucleotides required for amplification (Table 2). When genomic sequence was not available in the database, the cDNA sequence was used as primer template.

Genomic DNA was extracted from sperm cells from a single animal using the GenElute™ Blood Genomic DNA Kit (Sigma-Aldrich, St. Louis, MO, USA). Genomic DNA amplifications were conducted on an Eppendorf thermocycler as follows: 94 °C for 2 min, then 4 cycles of 94 °C for 1 min, 53–57 °C for 1 min (see Table 2), 72 °C for 5 min, then 30 cycles of 94 °C for 30 s, 53–57 °C for 30 s, 72 °C for 5 min, and 72 °C for 10 min, with Taq DNA Polymerase (Sigma-Aldrich). MT6 and MT7 gene amplicons were obtained amplifying two overlapping fragments (Up and Down).

Amplified genomic fragments were cloned in plasmid vector pGEM-T Easy and MT clones were fully sequenced by primer walking. Sequences were assembled with Codon Code aligner and were annotated using *with similarity* gene prediction programs and then manually curated.

cDNA and genomic sequences were compared using Wise2DBA (http://www.ebi.ac.uk/Tools/psa/wise2dba/) and polyadenylation signal in silico predictions were performed by Poly(A) Signal Miner, at http://dnafsminer.bic.nus.edu.sg/.

### 4.2. Sequences and Phylogenetic Analysis

MT sequences were retrieved from protein databases in NCBI or from the translated nucleotide database. Moreover, many searches both in Expressed Sequence Tag (EST) and Transcriptome Shotgun Assembly (TSA) databases, using TBLASTN software, were performed using both PlMTs and other MT amino acid sequences already annotated as queries. Pam30 matrix and no filter for simple sequences were used as parameters.

EST and TSA sequences were collected, translated using open reading frame (ORF) Finder at NCBI (https://www.ncbi.nlm.nih.gov/orffinder/) or the Translate tool at ExPASy (http://web.expasy.org/translate/) and tested for the presence of metallothionein domain (UniProt Knowledgebase).

MT protein sequences were aligned with T-Coffee software (http://tcoffee.crg.cat/apps/tcoffee/index.html) [72] and MSA was used to construct the phylogenetic tree by the neighbour-joining method with PHYLIP software (http://bioweb2.pasteur.fr/docs/phylip/doc/main.html), performing 1000 bootstrap replicates. The outgroup was *Tetrahymena pyriformis* MT-2 (ABF61447.1) [37], that we already used also in a previous work [25]. Phylogenetic trees were visualized with TreeDyn [73], and MSA was visualized with ESPript (http://espript.ibcp.fr/ESPript/cgi-bin/ESPript.cgi; [74]). MT sequence accession numbers used for alignments are listed in Table 1.

MT genomic sequences already annotated were retrieved from GenBank or from specialized databases. Unannotated MT genes were searched in whole genome shotgun (WGS) contigs or genome databases with BLAST (or BLAT), using transcript sequences as queries (see Table 1). The sequences were submitted to GenBank and will be provided during review.

### 4.3. Homology Modelling and Structural Characterization of PlMTs

The 3D structure of *P. lividus* homologues were reconstructed by homology modelling via the Phyre 2 software [75], with an intensive modelling mode as reported elsewhere [76,77]. Candidate structures for homology modelling were selected according to pairwise alignment. At least four different structures were used as templates for each generated structure, and homology models were built for all of the sets of proteins. Validation of the structural protein models was performed by assessing the Ramachandran plots. Cycles of clash minimization were also performed for the refinement of structures. Secondary structures assignments and relative solvent accessibility (RSA) were calculated by the DSSP program [78] as implemented in ENDscript, http://endscript.ibcp.fr [74].

### 4.4. Embryo Cultures, Metal Treatments and Morphological Analysis

Gametes were collected from gonads of the sea urchin *P. lividus*, harvested from the West Coast of Sicily. Eggs were fertilized and embryos reared at 18 °C in millipore-filtered seawater (MFSW) at the dilution of 5000/mL in glass beakers. In metal-exposure experiments, embryos were continuously cultured after fertilization in the presence of different metal concentrations (ZnCl_2_, NiCl_2_, PbCl_2_ serial dilutions in MFSW from 10^−4^ to 10^−8^ M, or CuSO_4_ from 10^−5^ to 10^−8^ M), and their development was monitored up to 48 h. For total RNA extraction and for WMISH, embryos were collected by low-speed centrifugation at 4 °C and processed as reported in the respective sections.

Morphological analysis of embryo development was performed after immobilization of embryos with 0.1% formaldehyde (final concentration) in seawater and observation under an Olympus microscope (OSP-MBI; Olympus, Tokyo, Japan).

### 4.5. RNA Extraction and RT-qPCR

Total RNA was extracted from fertilized eggs and from embryos at 4, 8, 12, 16, 20, 24, 31, 36 and 48 h of development in MFSW. Moreover, RNA was extracted also from metal-treated *P. lividus* embryos at the gastrula stage (24 h) with the RNeasy Mini Kit (Qiagen, Hilden, Germany) following the manufacturer’s instructions and performing DNase treatment. The RNA quality assessment, RT-qPCRs were performed as already described in Ragusa et al. [25]. Serial dilutions of cDNA (the standard curve method) permitted to calculate target cDNA starting quantity referred to 18S rRNA quantity in arbitrary units (considering 1 AU as 18S rRNA/1000).

### 4.6. Whole-Mount In Situ Hybridization

**Figure 14:**
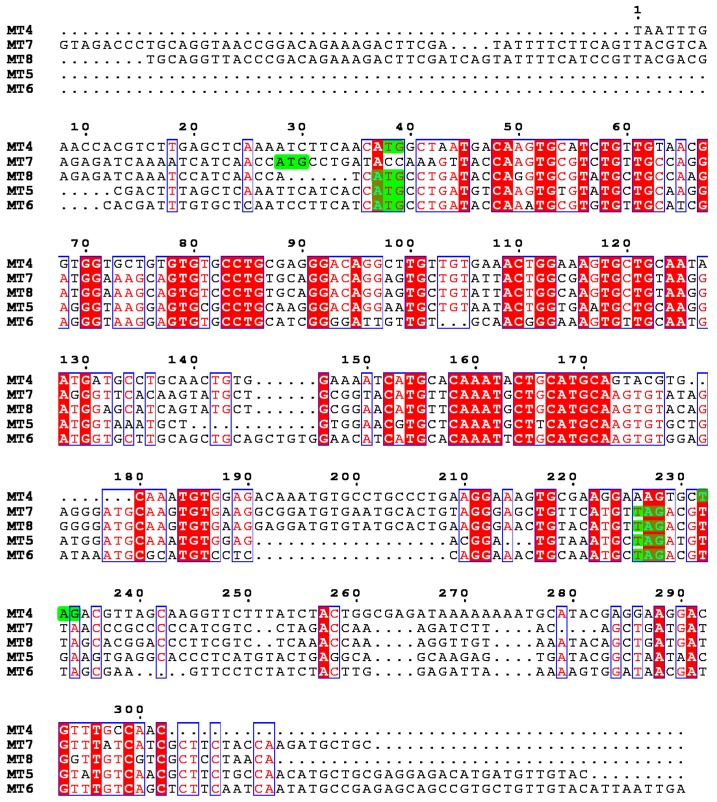
Multiple nucleotide sequence alignment of the probes used in WMISH experiments showing identities (red) and differences (black) among them. Red shading represents identity among all sequences. Dots denote gaps. Start and stop codons are in green.

Single strand probes were synthesized from cDNA *MT*s by asymmetric PCR [79,80] using the PCR DIG Probe Synthesis Kit (Roche). Primers used for probe amplification were designed from full-length coding sequences (Table 3). Probe sequences were aligned with Clustal Omega (http://www.ebi.ac.uk/Tools/msa/clustalo/) to show differences between them (Figure 14). Embryos were fixed in 2.5% glutaraldehyde solution on ice for 1–2 h [59]. Hybridization was performed at 42 °C (45 °C for MT8 probe) in hybridization buffer (50% formamide, 0.6 M NaCl, 5 mM EDTA, 20 mM Tris-HCl pH 7.5, 2× Denhardt’s, 500 µg/mL yeast tRNA, and 0.1% Tween-20). Probe concentrations were 0.5–1.0 ng/µL. Post-hybridization washes were: hybridization buffer, PBST (PBS, 0.1% Tween-20), 1× SSCT (1× SSC, 0.1% Tween-20), 0.5× and 0.2× SSCT, each 20 min at 60 °C (63 °C for MT8 probe). Subsequently, the antibody incubations were performed out at room temperature with 1:1000 diluted anti-DIG Fab-AP-conjugate (Roche, Basel, Switzerland). The embryos were extensively washed before staining reaction in PBST and twice with alkaline phosphatase (AP) buffer (100 mM Tris-HCl pH 9.5, 100 mM NaCl, 50 mM MgCl_2_, and 1 mM levamisole, 0.1% Tween-20). For staining, 5-Bromo-4-chloro-3-indolyl-phosphate (BCIP) and nitro blue tetrazolium were used. Controls were performed using sense probes (data not shown).

## Figures and Tables

**Table 1:** Species, protein, transcript and gene sequence IDs collected for phylogenetical and gene structural analyses.

Taxonomy	Species	Name	ID Protein/Reference	ID mRNA	ID Gene/Scaffold #
Echinoidea Echinoida	*Paracentrotus lividus*	PlMT4/8	Ragusa et al., 2013		§
Echinoidea Echinoida	*Sterechinus neumayeri*	SnMT	P55953		
Echinoidea Echinoida	*Strongylocentrotus purpuratus*	SpMTA	P04734.2	SPU_017989.3a	Scaffold1168 v3.1
		SpMTB2	Q27287	SPU_017134.3c	Scaffold1906 v3.1
		SpMTB1		SPU_001866.3a	Scaffold249 v3.1
Echinoidea Echinoida	*Heliocidaris tuberculata*	HtMT1	§	comp104953_c1_seq2 ◊	
		HtMT2		comp104953_c1_seq1 ◊	
Echinoidea Temnopleuroida	*Sphaerechinus granularis*	SgMTA	Q26497		
		SgMTB	Q26496		
Echinoidea Temnopleuroida	*Lytechinus pictus*	LpMT1	O02033		
Echinoidea Arbacoida	*Arbacia lixula*	AlMT1	§	c31462 _g1_i3 ¶	
		AlMT2		c31386 _g2_i2 ¶	
Echinoidea	*Echinarachnius parma*	EpMT	§	GAVF01002251	
Clypeasteroida					
Echinoidea Cidaroida	*Eucidaris tribuloides*	EtMT1	§	JI315060	§ JZLH010301553
		EtMT2		GAZP01041405	
		EtMT3		JI295076	
Asteroidea	*Asterias forbesi*	AfMT	§	GAUS01062044.1	
Asteroidea	*Asterias rubens*	ArMT	§	GAUU01048766.1	
Asteroidea	*Asterias amurensis*	AamMT	§	GAVL01015559.1	
Asteroidea	*Leptasterias* sp. *AR-2014*	LsMT	§	GAVC01041863	
Ophiuroidea	*Ophiocoma echinata*	OeMT1	§	GAUQ01108229	
		OeMT2		GAUQ01027563	
		OeMT3		GAUQ01073893	
Holothuroidea Aspidochirotida	*Apostichopus japonicus*	AjMT	§	GH551565	§ MODV01037111 +MODV01043468
Holothuroidea Aspidochirotida	*Parasthicopus parvimensis*	PpMT	§	Locus_1_Transcript_31588/53328	§ Scaffold4005 KN886207.1 + Scaffold3285 KN885487.1
Holothuroidea Aspidochirotida	*Parastichopus californicus*	PcMT1	§	GAVO01014408.1	
Holothuroidea Dendrochirotida	*Sclerodactyla briareus*	SbMT	§	GAUT01018048.1	
Crinoidea		-			
Hemichordata		-			
Urochordata Enterogona	*Ciona intestinalis*	CiMT1	ACN32211.2 Franchi et al., 2010	BW491384 FJ217357	Scaffold 186 (JGI database)
Urochordata Enterogona	*Ascidia sydneiensis samea*	AsMT	Yamaguchi et al., 2004	Asy-sig-715 and Asy-sig-997	
Urochordata Stolidobranchia	*Herdmania curvata*	HcMTA	§	AY314949	
		HcMTb		AY314939.1	
Urochordata Stolidobranchia	*Herdmania momus*	HmMT	§	EL733027.1	
Cephalochordata Amphioxiformes	*Branchiostoma floridae*	BfMT1	Guirola et al., 2012	BW764364	Guirola et al., 2012
		BfMT2_S		FE561990.1	
Cephalochordata Amphioxiformes	*Branchiostoma belcheri*	BbMT20	XP_019631158.1	XM_019775599.1	NW_017803933 (AYSS01018500)
Craniata Cyclostomata	*Lethenteron camtschaticum*	LcMT	§	DC612982.1	§ APJL01076593 + APJL01076594 + APJL01076595
Craniata Cyclostomata	*Petromyzon marinus*	PmMT	§	CO548937.1	
Craniata Gnathostomata Elopomorpha	*Anguilla anguilla*	AaMT	ABF50549.1	DQ493910.1	§ AZBK01727549
Craniata Gnathostomata Otomorpha	*Ictalurus punctatus*	IpMT	O93571	NM_001200077.1 or AF087935 and JT349175.1	NC_030419
Craniata Gnathostomata Otomorpha	*Clarias macrocephalus*	CmMT	AGC79138.1	JX312865.1	
Craniata Gnathostomata Otomorpha	*Danio rerio*	DrMTBl (like)	ENSDARP00000131449 (CAA65933.1)	ENSDART00000170342	ENSDARG00000102051
		DrMT2	ENSDARP00000061006 (AAH49475.1)	ENSDART00000061007	ENSDARG00000041623
Craniata Gnathostomata Euteleosteo-morpha	*Takifugu rubripes*	TrMT2	ENSTRUP00000022394	ENSTRUT00000022487	ENSTRUG00000008907.1
Craniata Gnathostomata Primates	*Homo sapiens*	HsMT1A	NP_005937.2	NM_005946	Gene ID: 4489

Scaffold #: Scaffold number. § Sequences identified in this work. ◊ Heliocidaris T_Trinity database. ¶ Available in Echinobase.

**Table 2:** Oligonucleotides used as primers for the genomic DNA amplifications.

Name	Forward Primer	Reverse Primer	Ta
MT4	TTGGTGATAATAAATGAACCTTGGAG	AGACTGGACAGTATCTAATTGGACAG	55
MT5	CAGACAGGTCTCCGTCTCGC	CGCTTCAAGCTCGCCACATT	57
MT6_UP	CACGGTGTTTTGGTTTGATGTC	TTTGTGCATGATGTTCCACAGC	57
MT6_DW	CACGATTTGTGCTCAATCCTTCAT	CAAGTGCTGGTTGCTATCCTG	57
MT7_UP	GCATGAGCAAAACCGTAGTCAG	ACATTCTGGATTCTTCTGCGTCG	53
MT7_DW	TAATGAAACCAGCCCACGATCA	TCACTCCGTCTTTGCAATCTT	57
MT8a	ATCAGTTACGACGAGAGATCAAATC	TTGGCTATCGCAAGACGTTC	57
MT8b	AAATGTAAATCAGTGACAGGACG	GAAACAATAAGTCATCAAATAACAAAAC	53
MT8c	CTTCTGAGTCTAGCGTTTCCTTGAAG	CTATCGCAAGACGTTCGTGAGC	55

Ta: annealing temperature. UP: up fragment amplification, DW: down fragment amplification.

**Table 3:** Primer sets for probe labelling (WMISH).

Probe	Forward (Sense) Primer	Reverse (Antisense) Primer
*MT4*	TAATTTGAACCACGTCTTGAGC	GTTGGCAAACGTCCTTCCTC
*MT5*	CGACTTTAGCTCAAATTCATCACCATG	GTACAACATCATGTCTCCTCGC
*MT6*	CACGATTTGTGCTCAATCCTTCAT	TCAATTAATGTAAAACAGCACGGC
*MT7*	GTAGACCCTGCAGGTAACCG	GCAGCATCTTGGTAGAAGCG
*MT8*	TGCAGGTTACCCGACAGAAAG	TGTTAGGAGCGACGACAACC

## Abbreviations

EST	Expressed sequence tags
MFSW	Millipore filtered sea water
MSA	Multiple sequence alignment
Myr	Million years ago
PlMTs	*Paracentrotus lividus* metallothioneins
PMCs	Primary mesenchyme cells
RSA	Relative solvent accessibility
RT-qPCR	Reverse transcription and quantitative (real time) PCR
SpMTs	*Strongylocentrotus purpuratus* metallothioneins
TSS	Transcription start site
WGS	Whole genome shotgun
WMISH	Whole-mount in situ hybridization
